# Accuracy of correction of a hexapod frame, patient-specific osteotomy and reduction guides, and hinged circular external fixation in a 3D-printed canine antebrachial deformity model

**DOI:** 10.3389/fvets.2024.1296371

**Published:** 2024-02-28

**Authors:** Neil J. Burton, Bill Oxley

**Affiliations:** ^1^Wear Referrals Veterinary Hospital, Part of Linnaeus Veterinary Limited, Bradbury, United Kingdom; ^2^VET3D, Kendal, United Kingdom

**Keywords:** hexapod frame, dog, corrective osteotomy, growth deformity, circular fixation

## Abstract

**Objectives:**

This study aimed to objectively define whether human hexapod fixation (Maxframe), with or without the use of 3D-printed positioning guides, can correct a canine antebrachial deformity with greater accuracy than the clinically established techniques of 3D patient-specific osteotomy and reduction guides (3D-PSORG) or hinged circular external skeletal fixation (CESF).

**Methods:**

CT of a canine antebrachium was manipulated to induce distal radial deformity of the valgus, external torsion, and procurvatum, each of magnitude 20^o^. Five experiments were performed to correct the deformity via a distal radial and ulna opening osteotomy using: (1) A 3D-PSORG with the application of a locking plate, (2) hinged CESF, (3) Maxframe standard protocol, (4) Maxframe applied with patient-specific positioning guides (PSPGs), and (5) Maxframe with frame adjustment calculated from post-application CT. Following correction, all constructs were optically scanned, and objective measurement of the correction achieved was performed.

**Results:**

No construct returned the distal bone segment to its preoperative position in all planes. Translational malalignment in the sagittal plane had the highest magnitude of error for all constructs, with the Maxframe standard protocol showing the greatest error. Maxframe (PSPGs) showed the minimum error of all constructs in the frontal and sagittal planes.

**Clinical significance:**

In this 3D-printed model of antebrachial deformity correction, the hexapod frame with the use of PSPGs achieved better accuracy than 3D-PSORG and hinged CESF and may be a technique of future interest and development in the management of canine antebrachial limb deformity.

## Introduction

Antebrachial limb deformities are the most common appendicular malformation in dogs (1), with premature radial or ulnar physeal closure due to hereditary or traumatic injury being the most frequent cause (2, 3). When lameness develops as a consequence of deformity, surgical correction may be indicated. Numerous surgical strategies have been described, including linear external skeletal fixation (ESF) (4), hybrid ESF (5), hinged (6), and bi-level hinged (7) Ilizarov circular external skeletal fixation (CESF), and internal fixation (8, 9). Recently, the utilization of both patient-specific 3D-printed osteotomies and reduction guides (3D-PSORG) (10) and titanium plates for internal fixation for deformity correction was described (11).

**Figure 1:**
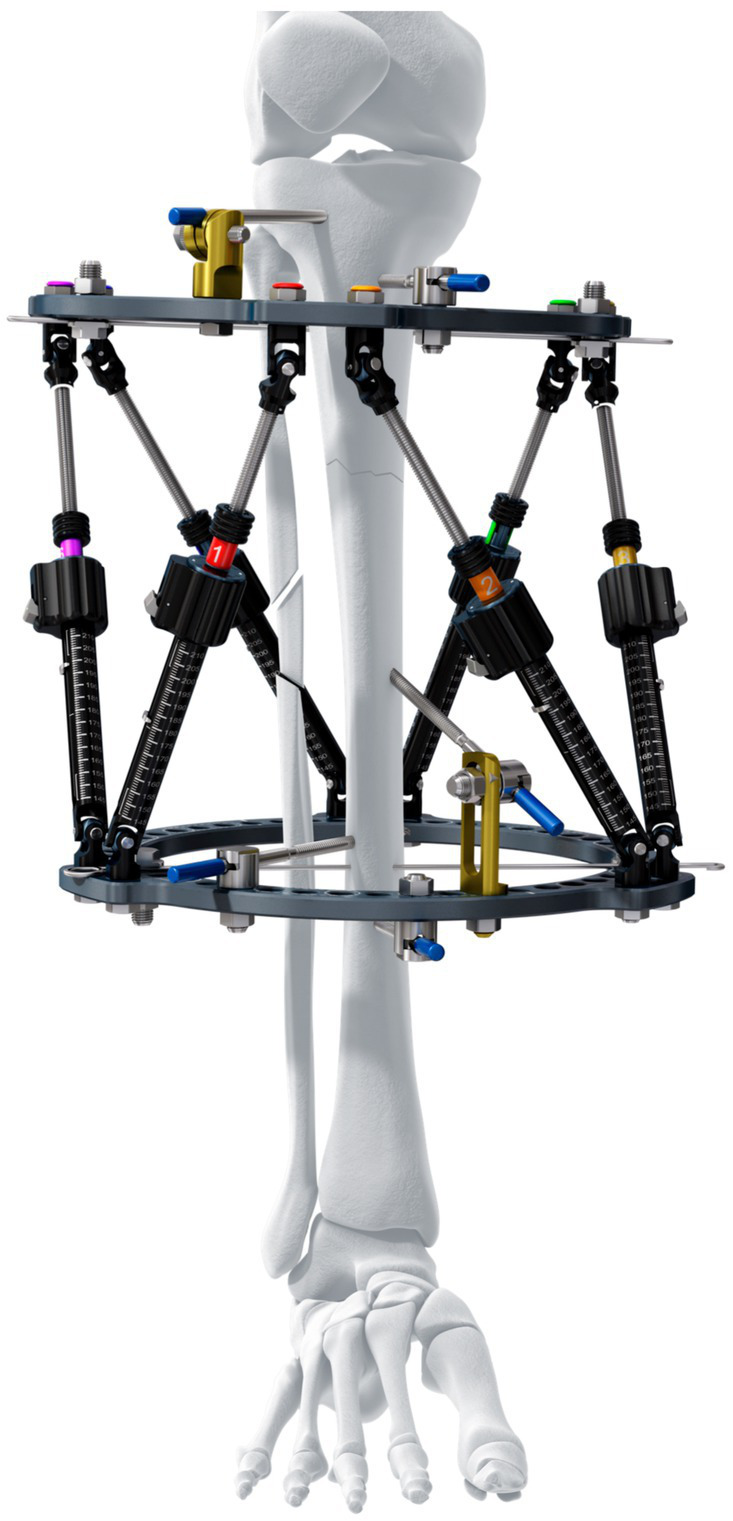
Maxframe™ is a human circular ESF two-ring hexapod system linked by six variable-length struts that permit six-axis correction with the use of computer software (reproduced with permission of DePuy Synthes).

The DePuy Synthes MaxFrame™ (Figure 1) is a human circular ESF two-ring hexapod system linked by six variable-length struts that permit six-axis correction with the use of computer software. The system is similar to that initially patented for human orthopedic use as the “Taylor Spacial Frame” by HS and JC Taylor in 1997 (12), with a design based on the Stewart Platform (13). Hexapod systems have been extensively used in human orthopedics over the last 25 years. The ability for simultaneous correction in all three planes, negating the need for sequentially hinged Ilizarov constructs, has proven precise and efficient for fracture repair (14), deformity correction, distraction osteogenesis (15, 16), and as an adjunctive aid for bone positioning prior to internal fixation (17).

Currently, there is no hexapod system for use in veterinary orthopedics. Such a system could permit more accurate correction of angular limb deformity in dogs than the techniques in use currently, but whether this would be the case or not is currently unknown. Correction of torsional deformity with veterinary CESF systems is currently limited to the fixed angular increment between adjacent ring holes. As such, the angle of torsional correction that can be achieved may not exactly match the degree of torsion present, predisposing to either under- or over-correction of deformity in this plane. The ability to correct in six planes with hexapod fixation can ensure that no residual translation or rotation exists. Studies evaluating the accuracy of complex lower-limb deformity correction in humans using hexapod fixation versus Ilizarov ring fixation have demonstrated much higher accuracy of correction of the former over the latter (15). Furthermore, the use of computer software to objectively plan strut adjustments negates human error in the subjective calculation of the adjustments to be made to correct the deformity. Minimally invasive application of external skeletal fixation may result in less soft tissue damage than open acute correction, and gradual resolution of the deformity through sequential strut adjustment permits safe distraction of neurovascular structures (18). Computer-aided design (CAD)-based surgical planning and 3D-printing of both patient-specific guides and patient-specific implants are established in veterinary orthopedics (19), and multiple reports document the accuracy of both deformity correction and implant placement using this technology (10, 20, 21). In human orthopedics, CAD-based planning and 3D-patient-specific guides (3D-PSGs) have also been used for deformity correction (22) and have recently been used to aid in the orientation of external fixation pin placement, which then sets the position of the subsequent external skeletal fixator construct on the bone (23).

The aim of this study was to objectively quantify the correction of a multi-planar canine antebrachial deformity in a 3D-printed bone model using a 3D-PSG, hinged CESF, and the Maxframe™ fixator applied with and without the aid of CAD-based surgical planning and patient-specific positioning guides.

Our hypothesis was that the Maxframe methodology could be successfully applied to correct the canine deformity with an accuracy of similar magnitude to the established techniques of 3D-PSG and hinged CESF already in use in veterinary orthopedics.

## Materials and methods

In order to assess the accuracy of the five different correction approaches detailed below, the study was designed such that each would be performed on an identical 3D-printed model of a canine antebrachial deformity in which the magnitude of the deformity in all planes was known. In this way, the accuracy of each approach could be quantified with reference to a known “gold standard” correction.

To identify a candidate antebrachium, the records of VET3D were searched for unilateral antebrachial deformity cases in large-breed, non-chondrodystrophic dogs. These were initially screened for the availability of medical records from the referring surgeon indicating clinical normality of the contralateral limb and the availability of CT DICOMs of that limb. These were further screened for appropriate quality for the creation of a 3D-printed model, for subjectively assessed normal antebrachial conformation, and for the absence of radiographic evidence of pre-existing joint pathology.

The case identified was a 2-year-old male neutered German shepherd dog who had been referred for CAD-based surgical planning and 3D-PSG design for the treatment of a left antebrachial deformity secondary to the partial premature closure of the distal radial physis. Both antebrachii had been CT scanned at the referring clinic with a 16-slice multi-detector CT scanner (Siemens, Somatom Scope, Erlangen, Germany).

**Figure 2:**
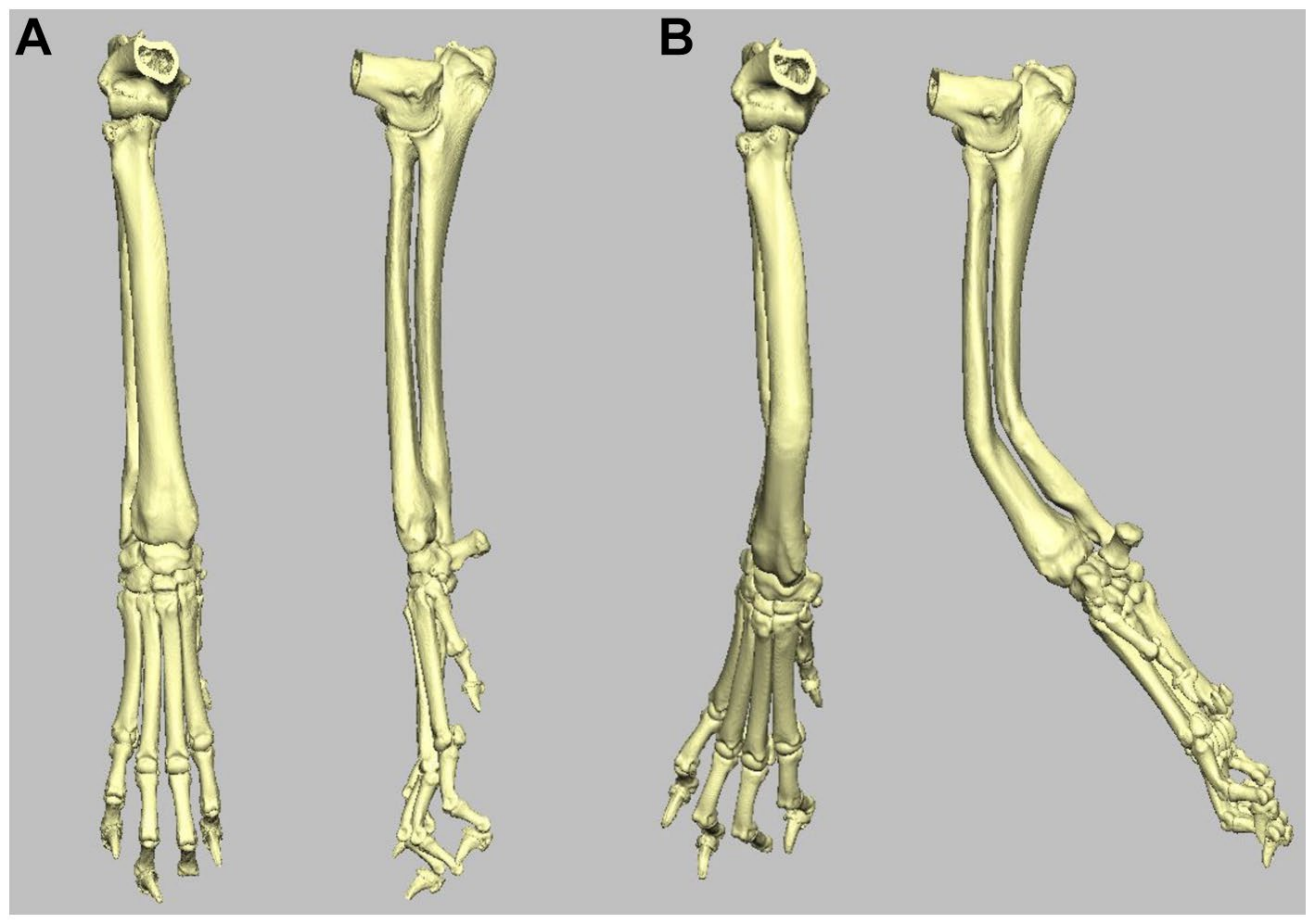
**(A)** Craniocaudal and mediolateral 3D volume render images of the right antebrachium used in the study. **(B)** Craniocaudal and mediolateral 3D volume render images of the same right antebrachium following inducement of 20^o^ distal valgus, procurvatum, and external rotation.

A surface-rendered representation of the right antebrachium was created with medical imaging software (Materialise Mimics, Leuven, Belgium) and exported to computer-aided design (CAD) software (Geomagics Freeform, 3D Systems, Rock Hill, United States), wherein the 3D model was manipulated to simulate a radial deformity. A virtual distal diaphyseal osteotomy was performed, and the distal segment was rotated by 20° in the frontal, sagittal, and dorsal planes to create valgus, procurvatum, and external torsion, respectively. The cortex was digitally reconstructed, resulting in a complete antebrachial model with a well-defined, uniapical, multi-planar deformity (Figure 2).

A copy of this model was 3D-printed for each of the five experiments in white methacrylate photopolymer resin using a Form 3 printer (Formlabs, Somerville, Massachusetts, United States). For those experiments requiring PSGs, these were printed using BioMed Amber resin on a Form 3B printer. BioMed Amber resin is certified as autoclavable and biocompatible (EN ISO 10993-1:2018; 10,993–3:2014; 10,993–5:2009) and is used for clinical 3D-PSG systems.

### Experiment 1: deformity correction with 3D-PSORG

CAD-based acute correction of the antebrachial deformity was planned using the aforementioned software. A virtual opening osteotomy was performed at the level of the frontal and sagittal plane centers of rotation of angulation (CORA). The distal segment was reorientated by 20° in the frontal, sagittal, and dorsal planes and was translated mediolaterally, craniocaudally, and proximodistal to appropriately align with the proximal antebrachial segment. An osteotomy and reduction guide system similar to those previously described (11, 24) was designed to facilitate this plan. This was 3D-printed along with a model of the post-correction antebrachium, the latter being used for the pre-contouring of a 12-hole 3.5 mm DePuy Synthes Vet 3.5 mm Locking Compression Plate (LCP) for application to the cranial radius.

**Figure 3:**
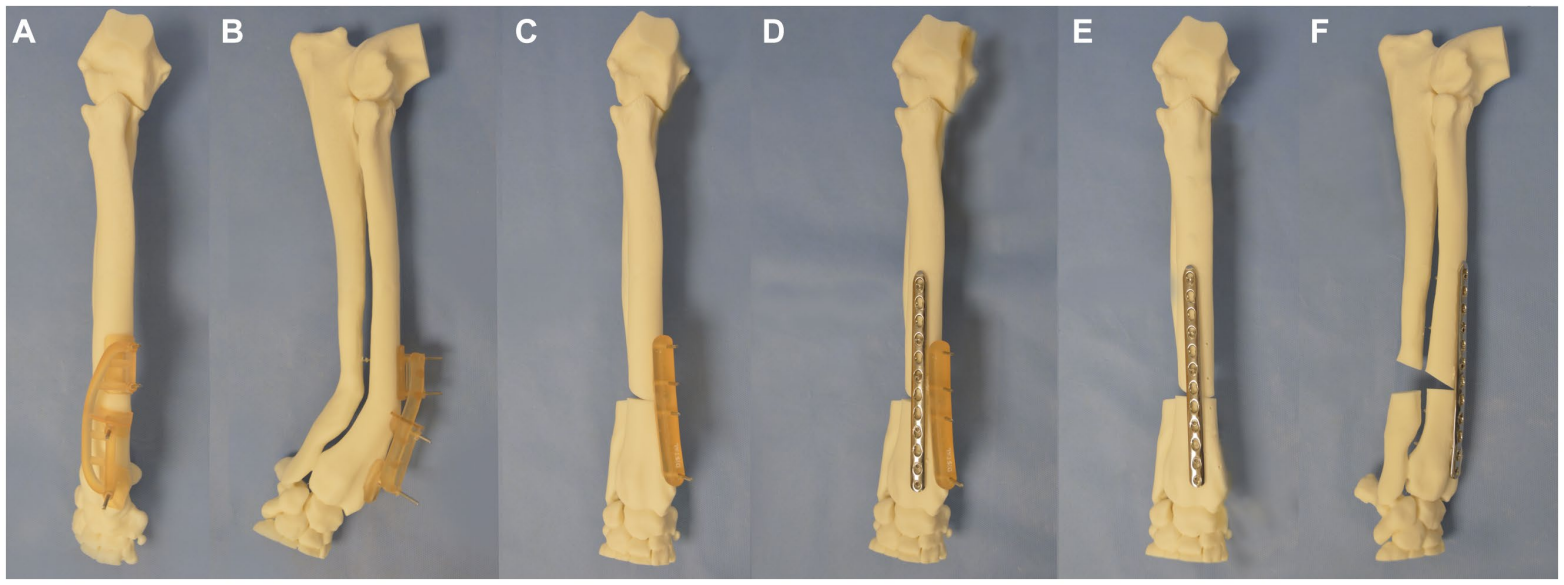
Experiment 1 **(A)** craniocaudal, **(B)** lateromedial: a patient-specific distal radial osteotomy guide was designed and attached to the radius with 4 K-wires, **(C)** a repositioning guide was used to reorientate the distal limb segment, **(D)** application of a pre-contoured LCP plate to the radius, **(E)** craniocaudal, and **(F)** lateromedial of antebrachium following opening osteotomy and fixation.

To perform the correction, the position of fit of the osteotomy guide on the craniomedial surface of the antebrachial model with the induced deformity was identified. The guide was attached to the model with four 2 mm Kirschner (K) wires, two proximal to the osteotomy guide plane and two distal to it (Figures 3A,B). Osteotomy of the radius was performed with a sagittal saw (DePuy Synthes UK, Colibri II) aligned to and in contact with the surface of the guide plane. A separate ulna osteotomy was performed, unguided, in the same plane as the radial osteotomy. Following the radial osteotomy, the osteotomy guide was removed, but the K-wires were left *in situ*. The reduction guide was applied to the K-wire pairs in each segment, aligning them in parallel. When the reduction guide was fully in contact with the surface of the model proximally and distally, the pre-planned relative orientation of the segments was achieved (Figure 3C). The pre-contoured 3.5 mm LCP plate was applied to the dorsal radius with the placement of six 3.5 mm locking screws using standard AO-ASIF techniques (Figure 3D). Thereafter, the removal of the four K-wires and reduction guide was performed (Figures 3E,F). The construct was then optically scanned (detailed below).

### Experiment 2: deformity correction with IMEX-hinged CESF

**Figure 4:**
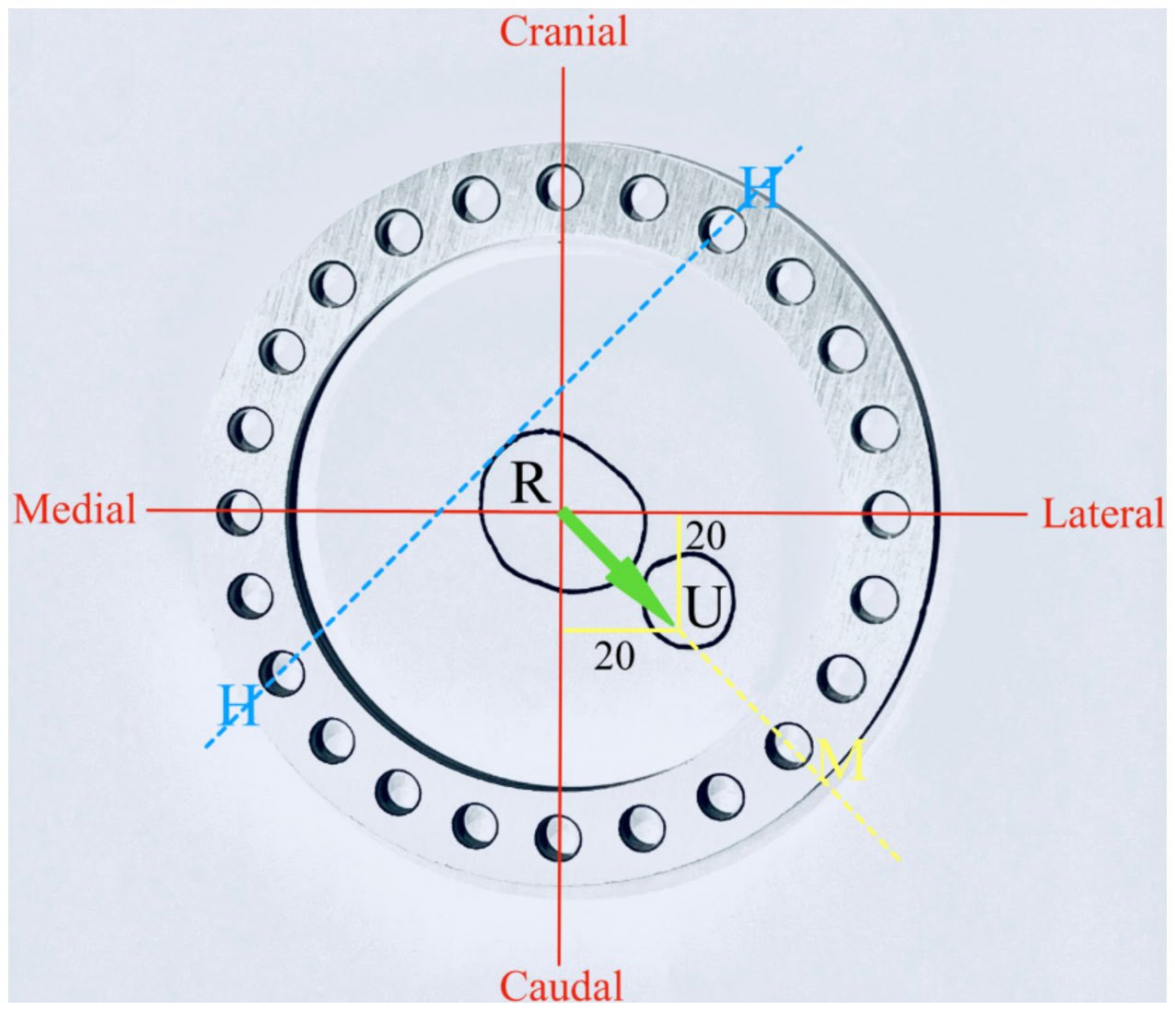
Experiment 2: planning for hinged IMEX fixator application R, radius; U, ulna; H, hinge; and M, motor. Twenty degrees of procurvatum and lateral deformity define the orientation of the motor and hinge position (see text for further details).

Correction of the deformity was planned with a three-ring hinged CESF using a previously described technique (6). Briefly, a three-ring hinged fixator was designed (IMEX Veterinary Inc., Texas, United States). Correction of the deformity was planned by drawing a cross-sectional diagram of the radius and ulna at the level of the deformity with overlay of the 20^o^ vector of valgus and procurvatum components of the deformity on the diagram (Figure 4). A ring size was chosen so as to anticipate sufficient clearance between the skin to mimic the selection that would be performed in a clinical case (118 mm ring with adjacent hole increments of 15^o^). The ring was overlaid on the diagram, and the hinge position was planned to align with the craniomedial aspect of the radius. A motor to permit an opening radial and ulnar osteotomy was positioned caudolaterally on the ring.

**Figure 5:**
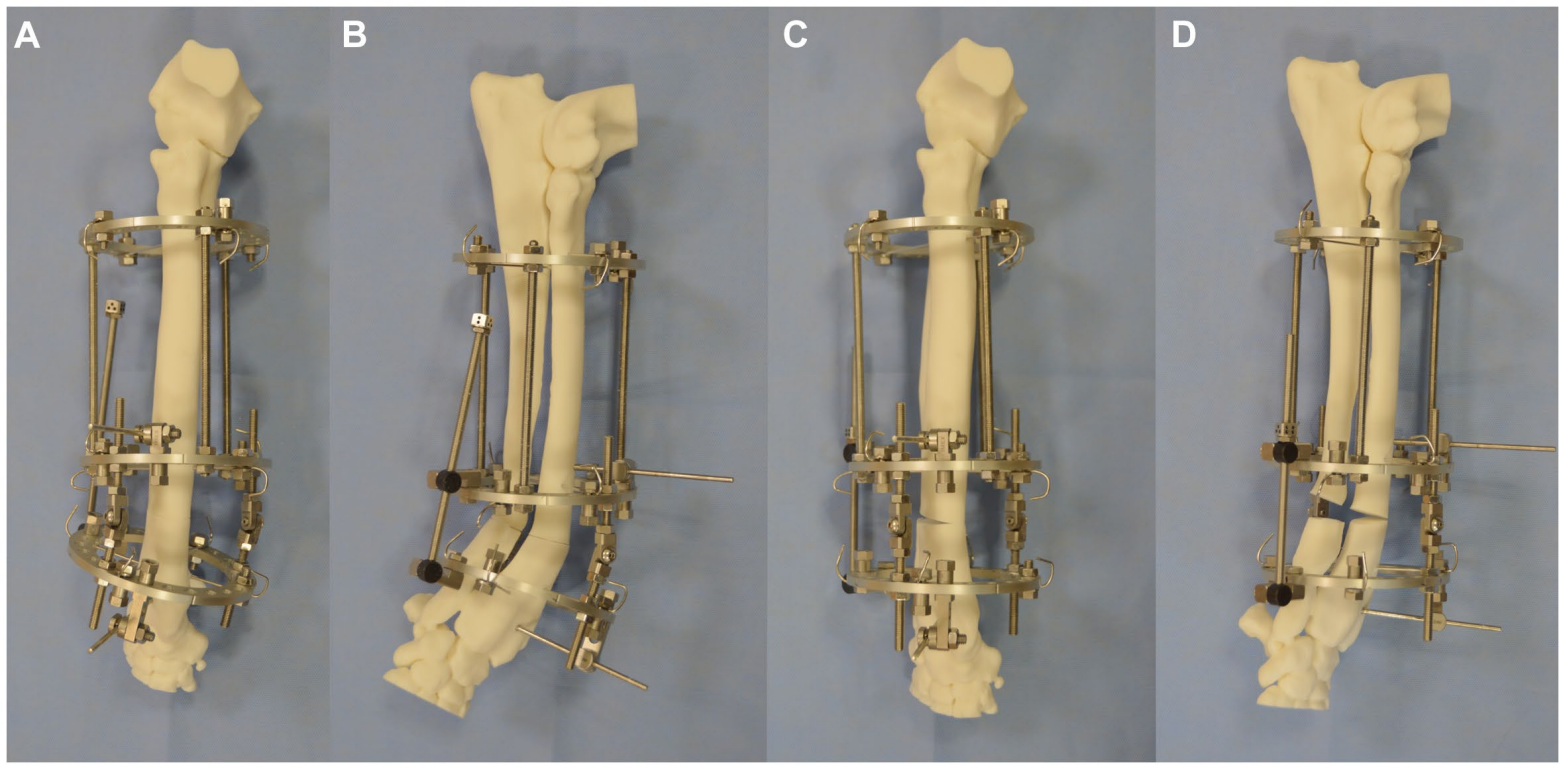
**(A)** Craniocaudal, **(B)** lateromedial of IMEX hinged fixator applied to the bone model with osteotomy having been performed. **(C)** Craniocaudal and **(D)** lateromedial of construct following the adjustment of the motor and radial and ulnar opening osteotomy.

The IMEX frame was then applied to the bone model via the application of two 1.8-mm Ilizarov wires per ring (Figures 5A,B). In addition, a 3.2-mm drop pin was applied to the middle and distal rings of the bone proximal and distal to the osteotomy for additional construct stability. Ilizarov wires were each tensioned to 95 kg with a Dyna Wire Tensioner (IMEX). Radial and ulnar osteotomies were performed at the level of the CORA. The construct was radiographed with the middle ring horizontal to the x-ray beam with a calibration marker (25 mm ball) and with both motors superimposed as previously described (6). The adjustment distance of the motor to resolve the deformity was calculated from the radiograph. The motor was then adjusted, resulting in an opening osteotomy of the radius and ulna being performed (Figures 5C,D). The distal ring was then detached from the middle ring and internally rotated relative to the middle ring by one hole, with subsequent reattachment to both hinges and the motor performed; this resulted in a 15^o^ reduction in the magnitude of the external rotational deformity. The construct was then optically scanned.

### Experiment 3: deformity correction with Maxframe™ standard protocol [frame adjustment calculated from post-application radiographs (XRs)]

A treatment plan was devised for the application of the hexapod frame to the bone model for deformity correction using the Maxframe™ User Interface (MUI) software. Both authors received online training from a DePuy Synthes representative on the use of the planning software and equipment prior to use.

**Figure 6:**
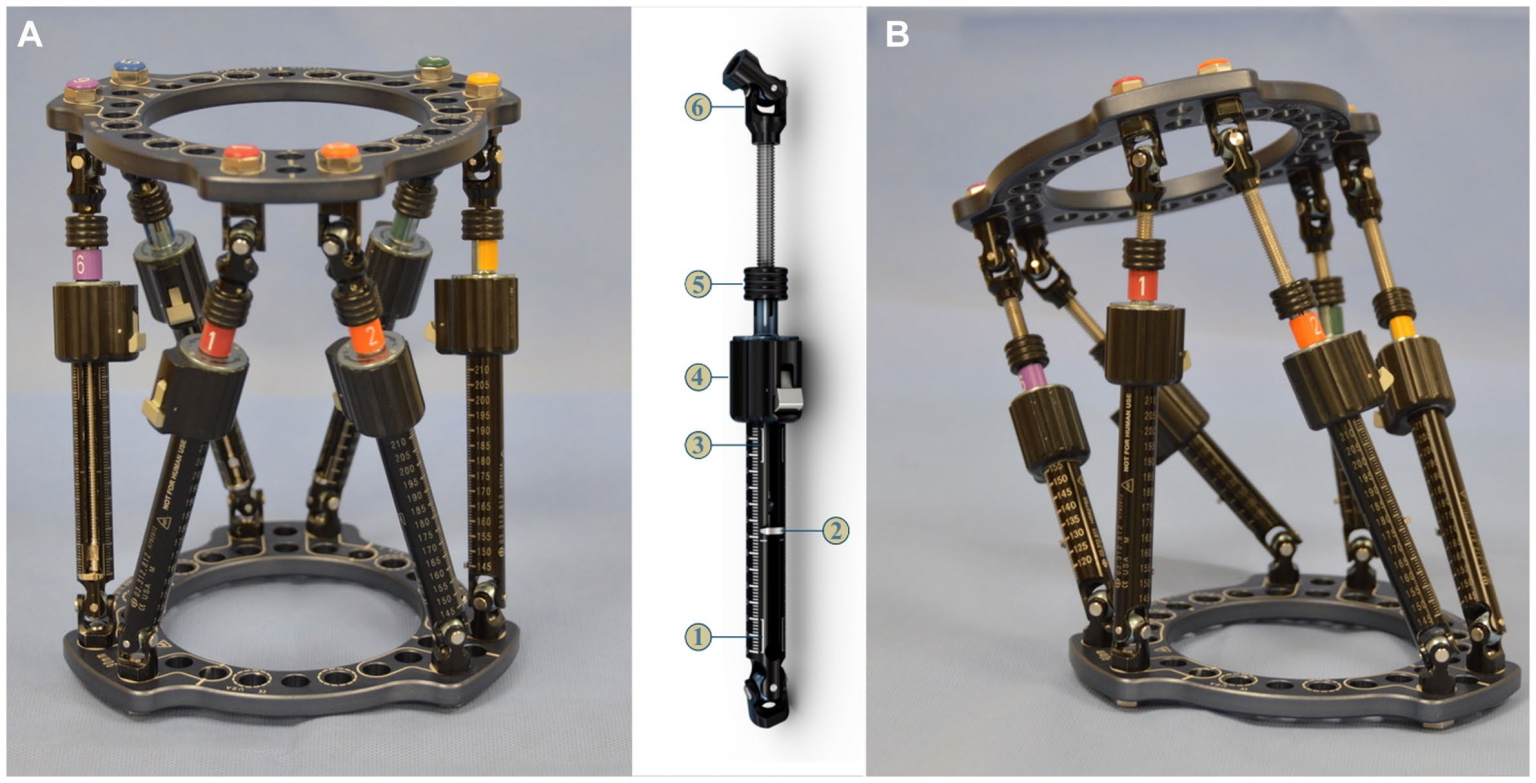
**(A)** Maxframe used for application to the bone model; strut identification numbers and colors were assigned by adding plugs and clips to the struts starting on the master tab on the proximal ring (1-red) and continuing anti-clockwise around the proximal ring, **(B)** quick adjust strut (QAS) 1—strut swap overlap line (bottom), 2—length indicator, 3—strut swap overlap line (top), 4—quick adjust locking collar, 5—adjustment knob, 6—spherical hinge, and **(C)** frame for application to the bone model following adjustment of the six QAS to align the proximal ring parallel with the radial head and distal ring parallel with the distal radial articular surface of the bone model.

Maxframe components were assembled with two full 90-mm diameter rings (Figure 6A); the ring size was chosen so as to anticipate sufficient clearance between the ring and skin to mimic the selection that may be performed in a clinical case. The rings were connected with six quick adjust struts (QAS) (Figure 6B). Strut identification numbers and colors were assigned by adding plugs and clips to the struts, starting on the “master tab” on the proximal ring, with strut one on the left of this tab, and continuing anti-clockwise around the proximal ring. The bone model was placed through the construct, and struts were then adjusted to subjectively align the proximal ring parallel with the proximal radial articular surface and the distal ring parallel to the distal radial articular surface, with the frame spanning the majority of the length of the radius. The length of each QAS with the frame rings locked in this position was recorded as referenced by the length indicator scale (in millimeters) on the side of each respective QAS (Figure 6C).

**Figure 7:**
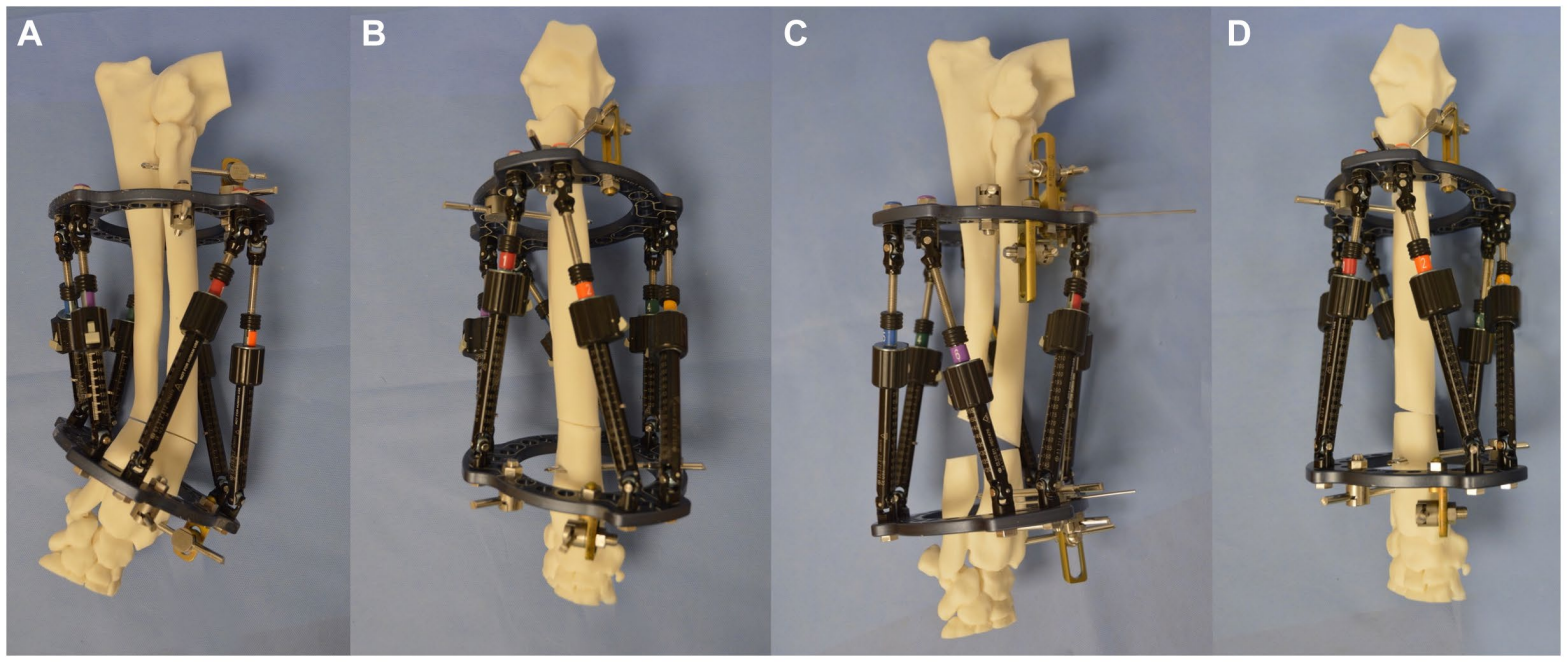
**(A)** Lateromedial, **(B)** craniocaudal images of the bone model with the Maxframe applied immediately following osteotomy, **(C)** lateromedial, and **(D)** craniocaudal images of the bone model following adjustment of the Maxframe and completion of deformity correction.

The Maxframe was then applied to the bone model using two 1.8-mm Ilizarov wires placed through the radius and attached to each respective ring, ensuring the proximal ring was aligned parallel with the humeroradial joint in both the craniocaudal and mediolateral planes (Figures 7A,B). A wire post, together with a clamp and subsequent application of a Schanz screw, was placed in the proximal and distal bone segments to provide additional construct stability. After the frame was mounted on the radius, osteotomies of the radius and ulna were performed at the level of the CORA as previously described. Craniocaudal and mediolateral radiographs referencing the proximal ring with the elbow were obtained for subsequent import into the MUI software.

**Figure 8:**
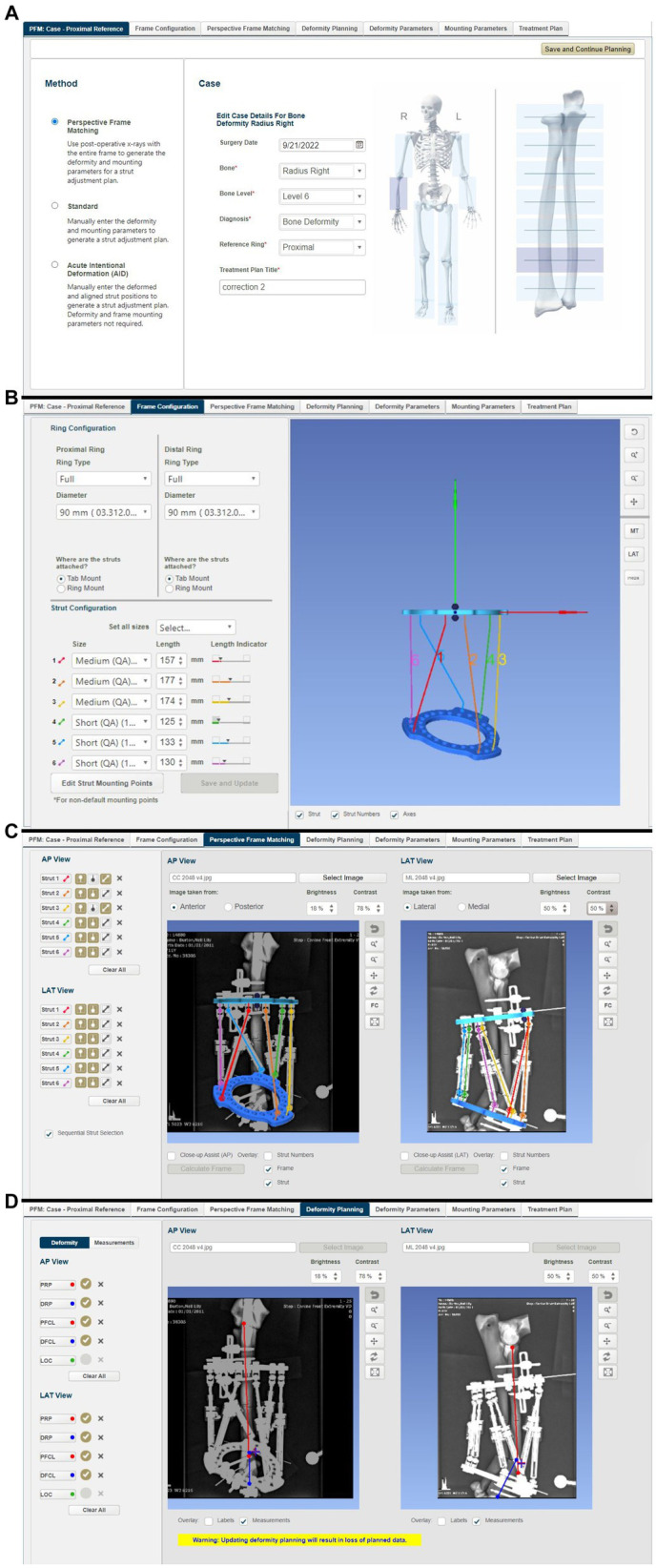
Summary of steps in the MUI software: **(A)** the bone to be corrected (radius) the deformity level (level 6 corresponding to the position of the CORA on the radius), and use of the proximal ring of the frame as a reference orthogonal to the long axis of the proximal antebrachium, **(B)** frame configuration was inputted (in this case that full 90 mm rings were used) and the lengths of all six quick adjust struts, as measured on the frame following application to the bone model but prior to osteotomy, **(C)** post-frame application radiographs were imported into the software. The location of the hinges at each end of all six struts was annotated on the radiographs allowing the software to calculate the ring positions, **(D)** proximal and distal referencing points (representing the planned points of contact between the proximal and distal segments after reduction), and the proximal and distal fragment center lines were overlayed on the radiographs in both planes.

A new treatment plan was created in the MUI software. Required input data included the bone being corrected (right radius), the deformity level (level 6 corresponding to the position of the CORA on the radius), and the use of the proximal ring of the frame as a reference orthogonal to the long axis of the proximal antebrachium (Figure 8A). The post-application radiographs were imported into the software as JPEG images. The perspective frame matching (PFM) option was selected, whereby the software uses additional inputted data regarding frame configuration and landmarks annotated by the surgeon on postoperative radiographs to calculate the magnitude of the deformity and the strut adjustment plan required to achieve the correction. Additional data required include frame configuration (in this case, full 90 mm rings were used) and the lengths of all six QAS’s on the frame when initially applied to the bone model (Figure 8B). Next, several landmark points and axes were annotated onto the post-application craniocaudal and mediolateral radiographs in the software. These included the locations of the hinges at each end of all six struts (Figure 8C), the proximal (PRP) and distal (DRP) referencing points (representing the planned points of contact between the proximal and distal segments after reduction), and the proximal (PFCL) and distal (DFCL) fragment center lines to define the deformity in both planes (Figure 8D).

**Figure 9:**
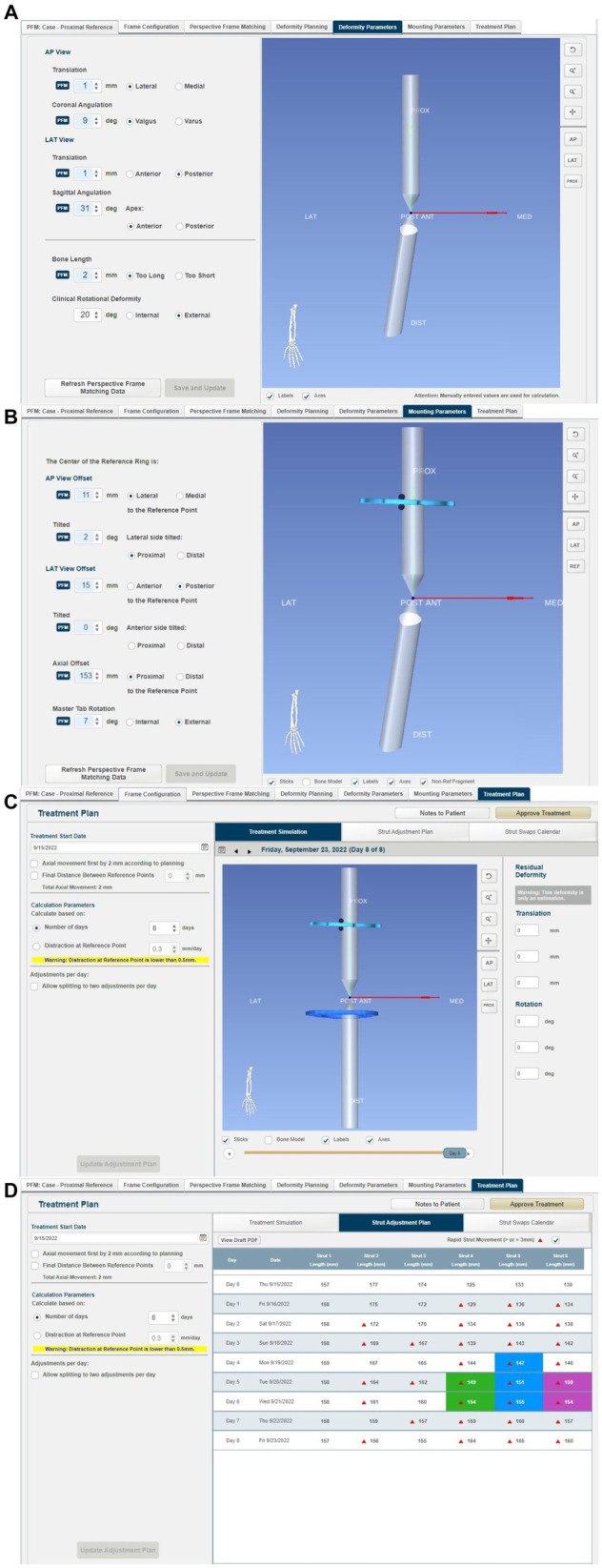
Summary of steps in the MUI software: **(A)** the MUI software calculated the deformity and frame mounting parameters. The deformity parameters described the relative angulation and translation between the proximal and distal segments in the frontal and sagittal planes, and any change in bone length. Torsional correction cannot be calculated by the MUI software and was manually entered, in this case 20^o^, **(B)** mounting parameters described the relative position and orientation of the Maxframe apparatus relative to the bone; these included the offset of the center point of the reference ring from the bone, and it is relative tilt, and the frontal, sagittal and dorsal planes, **(C)** review of the deformity correction—the sliding bar at the bottom of the screen permitted summary of daily adjustment until day 8 when correction of deformity was achieved, and **(D)** strut adjustment plan for each day of treatment coupled with days on when strut change from small to medium struts was required.

From this information, the MUI software then calculated the deformity and frame mounting parameters (Figures 9A–C). The deformity parameters described the relative angulation and translation between the proximal and distal segments in the frontal and sagittal planes and any change in bone length. The necessary dorsal plane (i.e., torsional) correction cannot be calculated by the MUI software and needs to be manually entered by the surgeon based on clinical assessment; in this case, the known 20^o^ value was entered. Mounting parameters described the relative position and orientation of the Maxframe apparatus relative to the bone; these included the offset of the center point of the reference ring from the bone, which is relative tilt, and the frontal, sagittal, and dorsal planes. Modification of these values was not necessary.

The MUI software then had the necessary information to calculate the strut adjustment plan (Figure 9D). The authors set the number of days for correction to eight, as this was the minimum timeline permitted by the software. Each QAS (1–6) was then sequentially adjusted for each day (0–8). The correction necessitated three “small” QAS to be changed for longer “medium” QAS on days 4–6 of the correction. Correction was performed acutely on the model by following the complete sequence of six strut adjustments calculated for 8 days in one session until all adjustments had been performed (Figures 7C,D). The construct was then optically scanned.

### Experiment 4: deformity correction with Maxframe^TM^ applied with patient-specific positioning guides designed based on CAD-based deformity correction planning incorporating frame adjustment calculation

A 3D representation of a 90 mm Maxframe ring with the strut hinges attached in the same standard positions used in the other experiments in this study was created from the optical scan data collected during experiment 3. Two copies were imported into the CAD software along with the virtual model of the deformed antebrachium. These were positioned and orientated proximally and distally as would be done clinically, just as performed when mounting actual frames on the bone models in experiments 2 and 3. The distances between the center points of the proximal and distal hinges for each strut were measured.

Next, the virtual osteotomy was planned and performed, and the distal segment was reorientated and reduced exactly as described in experiment 1. Importantly, the distal virtual CESF ring and associated hinges were moved identically to the distal segment so that their relative orientations were unchanged. The distances between the hinges for each strut were once again measured.

**Figure 10:**
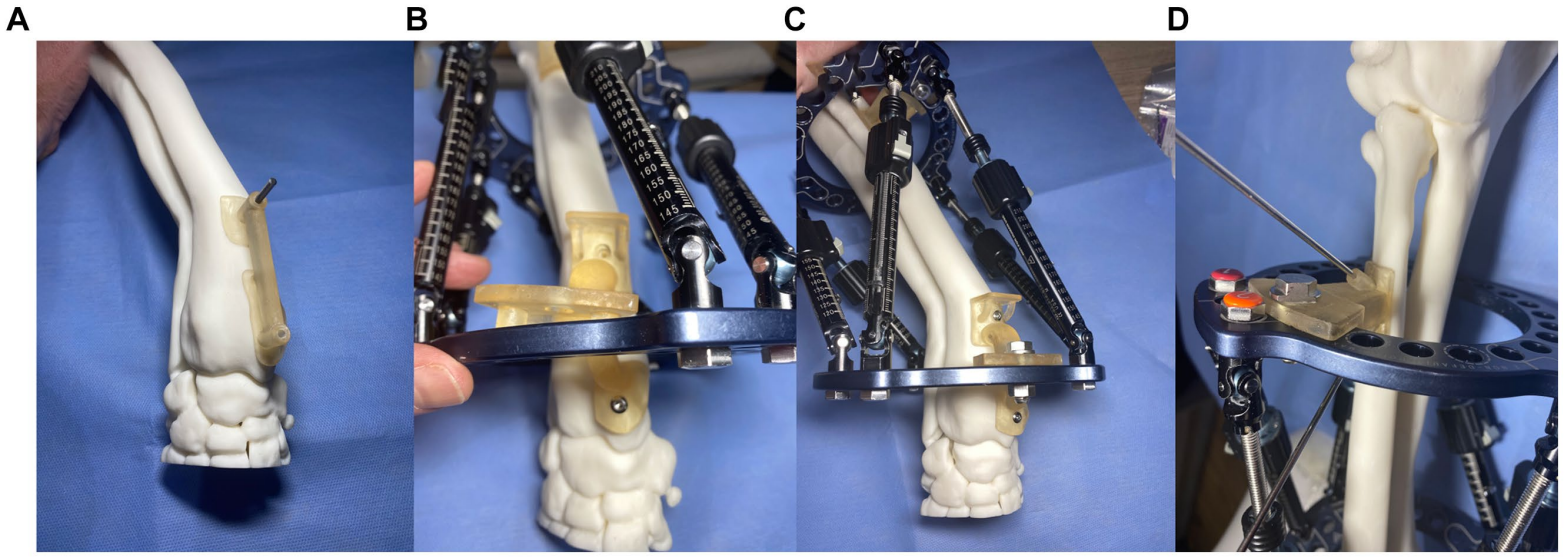
Experiment 4: Maxframe applied with patient-specific positioning guides to orientate the frame on the bone **(A)** first distal radial bone guide that positioned two 2.5 mm drill holes in the distal radius, **(B)** a second guide was attached to the bone through these two screw holes that incorporated an osteotomy guide plane and Maxframe mounting arm which attached to three specific holes on the distal ring, **(C)** following application of a nut and bolt through the central hole of the guide which locked the frame in position relative to the bone, and **(D)** third guide with identical frame mounting arm and two channels for fixation to the radius with two 2 mm Ellis pins.

The final step in the CAD was to design patient-specific frame positioning guides. These were necessary to translate the CAD-planned relative frame-antebrachial orientation to an identical real frame-antebrachial model orientation. Three small guides were designed. The first guide had two footprints that were mirrored representations of small areas of the craniomedial radial cortex distal to the CORA. This was designed such that the guide could fit only in a single location, thus accurately positioning two guide channels for 2.5 mm drill bits (Figure 10A). The second guide had similar footprints, countersunk screw holes for 3.5 mm cortical screws, an osteotomy guide plane, and a frame mounting arm that was attached to three specific holes on the distal CESF ring (Figure 10B). The point of attachment comprised two cylinders, which fitted into two holes in the ring, and a central hole where a bolt could be placed (Figure 10C). Once screwed to the radial cortex and bolted to the CESF, the guide provided robust temporary positioning as planned; however, some mild relative movement of the proximal ring was possible due to slight laxity in the strut hinges. To fix the exact planned position of the proximal ring relative to the bone, a third, smaller guide was designed. This had an identical frame mounting arm to the second guide and a small footprint with channels for two 2 mm Ellis pins (Figure 10D). The frame was attached to the model using Ilizarov pins and Schantz screws as described for experiment 3. The osteotomy was performed using an oscillating saw aligned with the guide plane of the distal guide. Finally, the two positioning guides were unbolted from the frame, the securing pins or screws were removed from the bone model, and the positioning guides were removed from the construct.

**Figure 11:**
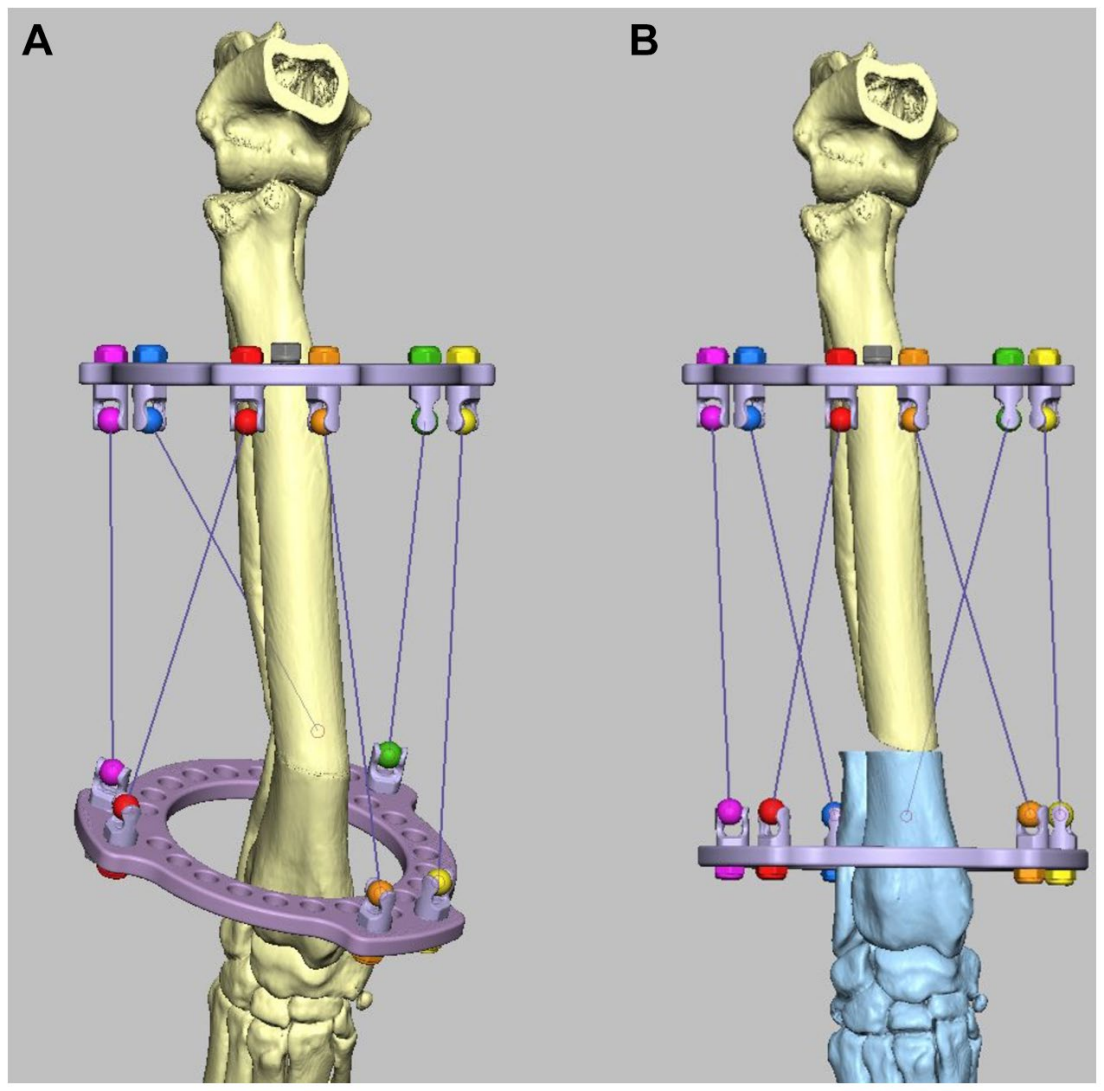
Experiment 5: Maxframe applied with frame adjustment calculated from post-application CT. **(A)** Virtual model of the Maxframe apparatus and the deformed antebrachium were created from the CT and imported into the CAD software and **(B)** the distal segment was reorientated and reduced and the distal virtual CESF ring and associated hinges moved identically to the distal segment so that their relative orientations were unchanged. The MUI software calculated the necessary strut adjustment plan using the acute intentional deformation (AID) function using the initial strut lengths measured from the applied apparatus, and the virtual post-correction strut lengths measured in the CAD.

The MUI software was used to calculate the necessary strut adjustment plan using the acute intentional deformation (AID) function. The data required were a description of the proximal and distal rings being used (90 mm full rings), the location of the strut hinges, and the starting and finished lengths of the struts as modeled in the CAD (Figures 11A,B).

As described for experiment 3, the strut lengths were adjusted according to the adjustment plan until the correction was completed. The construct was then optically scanned.

### Experiment 5: deformity correction with Maxframe^TM^ with frame adjustment calculated from post-application CT

The Maxframe apparatus was assembled and applied to a model of the deformed antebrachium, and freehand distal radial and ulnar osteotomies were performed in exactly the same way as described for experiment 3. However, rather than post-application radiographs being obtained, the construct was CT scanned. The length of each QAS was recorded. Virtual models of the Maxframe apparatus and the deformed antebrachium were created from the CT and imported into the CAD software; importantly, their relative “as-scanned” orientation was preserved (Figure 11A). Next, the distal segment was reorientated and reduced as previously described; as in experiment 4, the distal virtual CESF ring and associated hinges were moved identically to the distal segment so that their relative orientations were unchanged. The distances between the hinges for each strut were once again measured (Figure 11B).

The MUI software was used to calculate the necessary strut adjustment plan using the acute intentional deformation (AID) function using the initial strut lengths measured from the applied apparatus and the virtual post-correction strut lengths measured in the CAD. The strut lengths were adjusted according to the adjustment plan until the correction was completed, and the construct was optically scanned.

### Optical scanning

For each experiment, the corrected bone model and associated fixation devices were scanned using a Steinbichler/Zeiss Comet L3D_5M optical scanner scanned with a 500 mm field of view lens.^1^ Optical scanning was performed by the projection of a set of contrasting stripes on each construct surface with the use of deformation of the stripes to produce a point cloud. These points in 3D space were used to triangulate and produce mesh data with a mesh point spacing of 18 microns. Colin3D_2.1.0 software was used to stitch data to form an .stl file. In the case of the external fixation components applied in experiment 2 and the Maxframe construct in experiments 3–5, external fixation frames were digitally subtracted from the scanned mesh to leave the corrected bone model only for subsequent analysis.

### Measurement of deformity correction

**Figure 12:**
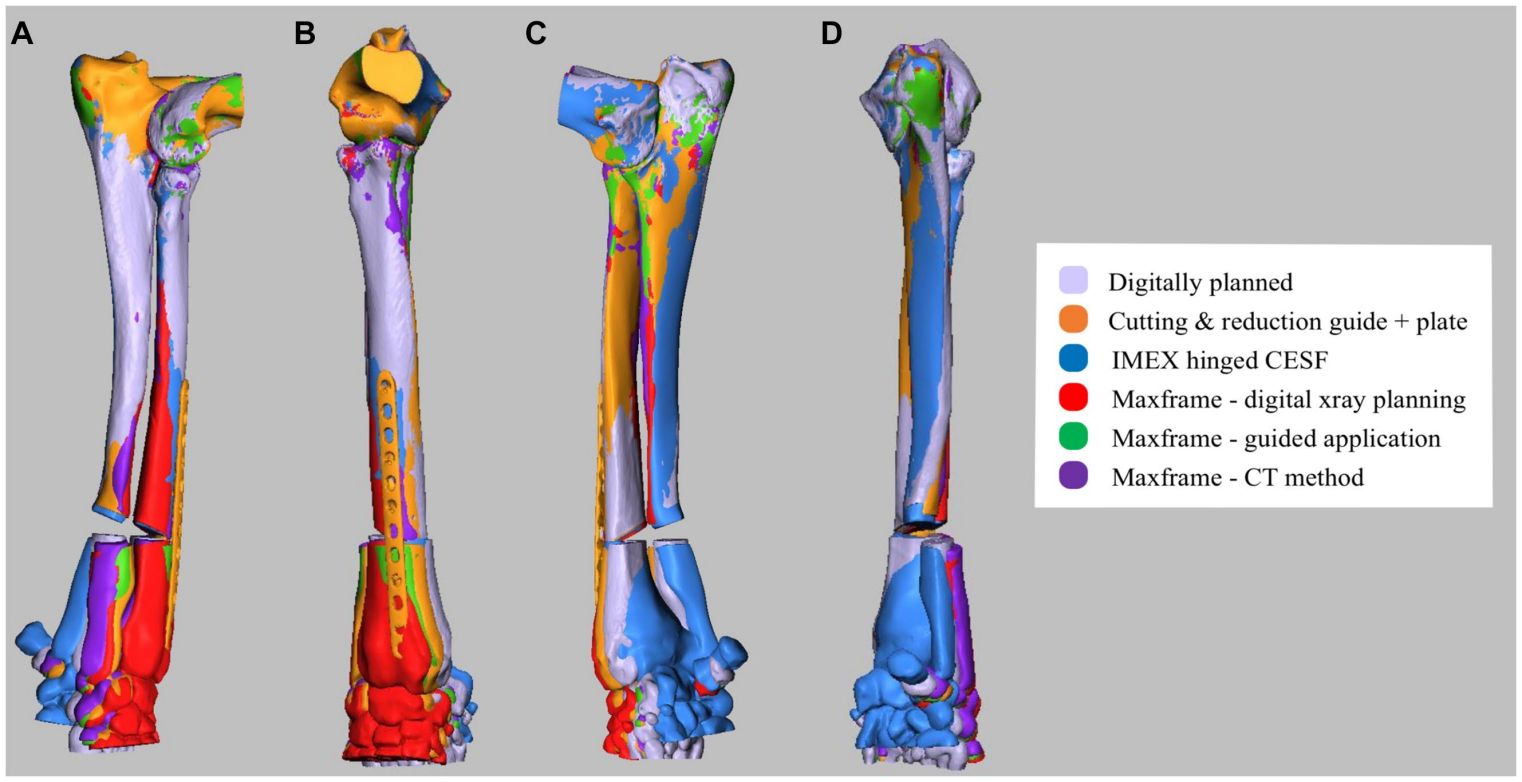
**(A)** Lateral, **(B)** cranial, **(C)** medial, and **(D)** caudal composite CAD 3D image showing the positioning of the antebrachium following opening osteotomy and planned adjustment versus the adjustment achieved for experiments 1–5.

The post-reduction optical scan data for each of the five experiments were used to create virtual models of the corrected antebrachii. The proximal portions of these models were identical to those of the original antebrachium prior to manipulation in CAD to induce the deformity and thus could be exactly orientation-matched by the CAD software using iterative closest point (ICP) analysis. As such, any difference between the original antebrachium prior to inducing the deformity and the achieved orientations of the distal segments after each surgery represented correction inaccuracy (Figure 12). This was quantified by using the same ICP analysis to measure the necessary angular and translational transformations required to match the orientations of the distal segments. This could be rigorously determined in all six degrees of freedom (three angular planes and three translational planes).

Angular transformations were determined based on the ICP analysis of the whole distal segment, with the exception of the proximal segment immediately adjacent to the osteotomy, which varied due to the differing freehand versus guided osteotomy positions and orientations. Translational transformation quantification required the planned versus achieved position of a specified point on the distal segment to be measured. Consistent proximal and distal reference points (i.e., the planned points of contact between the proximal and distal segments after reduction) were not present in all experiments due to the variability of osteotomy positions and orientations. Therefore, the lowest and most distal point of the extensor carpi radialis groove was specified, as it was considered that this would give a clinically relatable indication of translation inaccuracy.

## Results

For all five experiments, post-correction antebrachial alignment visually appeared subjectively appropriate. The angular and translational malalignments of the post-correction distal segment relative to the planned orientation, as measured on ICP analysis, are presented in Table 1. None of the constructs used returned the distal antebrachial bone model segment precisely to its preoperative position in all planes. Translational malalignment in the sagittal plane was consistently the highest magnitude of error for all constructs, with the Maxframe standard protocol showing the greatest error. Maxframe (PSPGs) showed the minimum error in frontal, sagittal, and dorsal plans of all constructs.

**Table 1:** Summary of angular and translational malalignments of the post-correction distal bone model segment relative to the planned orientation.

		Experiment 1 3D-PSORG/plate	Experiment 2 IMEX CESF	Experiment 3 Maxframe (XR)	Experiment 4 Maxframe (3D-PSG)	Experiment 5 Maxframe (CT)
Angulation malalignment relative to planned (degrees)	Frontal Positive = valgus Negative = varus	1.1	2.2	0.5	0.6	0.8
	Sagittal Positive = procurvatum Negative = recurvatum	−3.4	9.5	−6.4	−2.1	−2.3
	Dorsal Positive = internal torsion Negative = external torsion	3.5	−7.9	−6.8	−1.3	−1.1
Translational malalignment relative to planned (millimeters)	Frontal Positive = medial Negative = lateral	−3.7	−2.3	−7.8	−4.6	−4.9
	Sagittal Positive = caudal Negative = cranial	−8.1	9.5	−12.1	−7.3	−6.3
	Dorsal Positive = proximal Negative = distal	−2.1	1.4	−3.0	−2.9	−0.4

3D-PSORG, 3-dimensional patient-specific osteotomy and reduction guide; CESF, circular external skeletal fixator; XR, digital radiograph; 3D-PSG, 3-dimensional patient-specific guide; CT-computed tomography.

## Discussion

None of the constructs evaluated returned the distal antebrachial bone model segment to its exact preoperative position in all planes. Residual external torsional malalignment was greatest with the IMEX construct. Residual external torsion was predicted to be present in the IMEX experiment, as the correction of the torsional component of the deformity was constrained by the angle between adjacent holes in the ring. In the case of the 118 mm IMEX ring with 15^o^ increments, internal rotation of the distal bone segment by one hole (equivalent to 15^o^) was performed, thus anticipating of a residual external torsional deformity of 5^o^. A residual external torsion of 7.9^o^ was achieved. It is unclear how this small discrepancy between anticipated and measured torsional deformities developed. As the osteotomy was performed freehand, if this was mildly eccentric from the CORA, then the anticipated correction in all three plans would be altered, and our results would be predicted to show residual deformity in all three planes, as was the case. Equally, the position of the pre-fabricated frame on the bone was subjectively defined as described clinically (6), with the hinges positioned approximately level with the mediolateral component of the deformity. In the Marcellin-Little et al., study (6), residual mediolateral or craniocaudal deformity of magnitude 0–9^o^ was variably present postoperatively, although details on the magnitude of any residual rotational component of the deformity are not available.

Translational malalignment in the sagittal plane was consistently the highest magnitude of error for all experiments, with the Maxframe XR showing the greatest error. This was caudal in all experiments, bar the IMEX CESF. Of all experiments, Maxframe 3D-PSPGs showed the least error in the frontal and sagittal planes, and the error was of similar magnitude to that of Maxframe-CT in the dorsal plane. As such, we can accept our hypothesis that the use of Maxframe (specifically with the use of 3D-PSPGs) permitted correction of this canine antebrachial deformity with an accuracy of similar magnitude to the established techniques of 3D-PSG and hinged CESF.

A limitation of non-CAD-based deformity correction planning is that the deformity magnitude in each plane has to be calculated from 2-dimensional imaging. In the case of deformity correction with CESF, similar to experiment 2, correction has typically been calculated from measurement from radiographs, with an assessment of the magnitude of the torsional component of the deformity based on measurement from the patient (6). Similarly, in experiment 3 with the Maxframe, correction was calculated by the annotation of radiographs using the software. The accuracy of planning in this way is affected by variations in the appearance of a 2D-reference line drawn in any given plane due to associated deformities in the other two planes (25). When the magnitude of the deformity is small, it is anticipated that the error in the calculation may also be small. However, with more severe deformities that may often be encountered in veterinary orthopedics, errors in the calculation of the magnitude of the deformity may make the calculated values less reliable. In addition, identifying the specific landmarks required (e.g., joint angle, hinge/strut/ring position, and orientation) to facilitate calculation on a radiograph when there is component and bone superimposition can be challenging. CAD-based planning circumvents these difficulties as the entire process is performed in 3D.

This study has demonstrated that the use of the Maxframe can be integrated with 3D planning either via the use of preoperative CT or via the substitution of post-Maxframe application radiography for CT. The former permits accurate modeling of an optimal osteotomy position and orientation but requires the 3D relationship of the Maxframe apparatus to the bone to be known in order for the software to be able to calculate the necessary strut adjustments for the required correction. In the latter approach, the relationship between the Maxframe apparatus and bone is defined, and so the requirement for guided apparatus application is avoided. However, the disadvantage of this technique is that the osteotomy must be performed freehand. This is unlikely to be a significant problem in cases where the position and trajectory of the optimum osteotomy are easily identifiable (e.g., a well-defined uniapical CORA as in this experimental model). However, with a more complex correction osteotomy, if osteotomy is erroneously performed at a level different from the CORA, then Paley’s osteotomy rule 3 applies, and as such, co-linearity of bone is not maintained with iatrogenic translation as a consequence (26).

There are limitations to the study performed, and the results should be interpreted in this context. First, the experiments performed were on 3D-printed models rather than clinical patients, and as such, soft tissue structures influencing the placement of implants and the adjustment of the bone were not present. Second, no conclusions regarding bone healing with the use of a hexapod construct in dogs, when compared to the other constructs used, can be performed at this time. The Maxframe system is not licensed for use in canine patients, and as such, the assessment of its efficacy in the treatment of antebrachial deformity *in vivo* has not currently been explored. Third, due to cost constraints relating to optical scanning, it was not possible to repeat each experiment multiple times to assess for any repeatability error. However, no error in the application of the apparatus used in each experiment was appreciated by the authors, and the CAD methodology used for the measurement of the deformity corrections achieved was objective and of high accuracy for the adjustments performed in all three planes.

We used the pediatric Maxframe components in this study, which were appropriate for the antebrachial model chosen from a German Shepherd dog. The use of a dedicated hexapod system in canine patients would require the manufacture of struts and rings of smaller components in order to treat the range of patients that may present with limb deformities. The development of such components and canine-specific software for surgical planning would also have to not be prohibitively expensive in order for such a system to be implemented clinically. Initial training was required on the use of both the frame components and computer software for correction planning, representing a time investment. However, the technical support required thereafter was minimal. Subjectively, the time taken for the application of the Maxframe and IMEX hinged CESF to the bone models and their subsequent adjustments were comparable.

In summary, the results of this 3D-printed bone model of canine antebrachial correction suggest that hexapod frame correction with the use of PSPGs, when compared to the established clinical techniques of 3D-PSORG and hinged circular fixation, has accuracy sufficient to be an area of further research and potential clinical application in veterinary orthopedics.

## Data availability statement

The raw data supporting the conclusions of this article will be made available by the authors, without undue reservation.

## Author contributions

NB: Conceptualization, Investigation, Methodology, Visualization, Writing – original draft, Writing – review & editing. BO: Investigation, Methodology, Resources, Software, Validation, Visualization, Writing – original draft, Writing – review & editing.

## Footnotes

^1^CBM - Wales Centre of Advanced Batch Manufacture, IQ Building, Kinds Road, Swansea SA1 8EW, United Kingdom.
